# Radiation exposure and fluoroscopically-guided interventional procedures among orthopedic surgeons in South Korea

**DOI:** 10.1186/s12995-020-00276-x

**Published:** 2020-08-11

**Authors:** Seonghoon Kang, Eun Shil Cha, Ye Jin Bang, Teresa W. Na, Dalnim Lee, Sang Youn Song, Won Jin Lee

**Affiliations:** 1 Department of Occupational and Environmental Medicine, COMWEL Ansan Hospital, Korea Workers’ Compensation & Welfare Service, Gyeonggi-do, South Korea; 2 Department of Epidemiology and Health Informatics, Graduate School of Public Health, Korea University, Seoul, South Korea; 3 Department of Preventive Medicine, Korea University College of Medicine, 73, Goryeodae-ro, Seongbuk-gu, Seoul, 02841 South Korea; 4 Department of Policy and Administration, National Radiation Emergency Medical Center, Korea Institute of Radiological and Medical Sciences, Seoul, South Korea; 5 Department of Radiation Effects Research, National Radiation Emergency Medical Center, Korea Institute of Radiological and Medical Sciences, Seoul, South Korea; 6 Department of Orthopedic Surgery, Gyeongsang National University Hospital, Jinju, South Korea

**Keywords:** Fluoroscopy, Health professional, Occupational exposure, Orthopedics, Radiation

## Abstract

**Background:**

The use of fluoroscopically-guided interventional (FGI) procedures by orthopedic surgeons has been increasing. This study aimed to investigate the occupational radiation exposure among orthopedic surgeons in South Korea.

**Methods:**

A nationwide survey of orthopedic surgeons was conducted in South Korea in October 2017. The dosimetry data of the participants were obtained from the National Dosimetry Registry. The orthopedic surgeons were categorized by job specialty [spine or trauma specialists, other orthopedic specialists, and residents], and descriptive statistics for the demographics and work-related characteristics were presented. Multivariable logistic regression analysis was used to evaluate the risk factors for the orthopedic surgeons who were not linked with the dosimetry data.

**Results:**

Among the total participants (*n* = 513), 40.5% of the orthopedic surgeons spent more than 50% of their time working with the FGI procedures when compared with their overall work. The average frequency of the FGI procedures among the orthopedic surgeons was 12.3 days per month. Less than 30% of the participants were regularly provided with radiation monitoring badges. The proportion of subjects who always wore lead aprons and thyroid shields were 52 and 29%, respectively. The residents group experienced more unfavorable working conditions of radiation exposure than the other specialists. The dosimetry data were not significantly linked among the residents (odds ratio [OR] 2.10, 95% confidence interval [CI] 1.11–3.95) and orthopedic surgeons working at small hospitals (OR 4.76, 95% CI 1.05–21.51).

**Conclusions:**

Although orthopedic surgeons often performed FGI procedures, they wore protective gear less frequently, and a large proportion of orthopedic surgeons were not monitored by the national radiation dosimetry system. As the number of radiation procedures performed by the orthopedic surgeons increases, more intensive approaches are needed to reduce radiation exposure, especially for spine and trauma surgeons.

## Background

With the markedly increased use of fluoroscopically-guided interventional (FGI) procedures during surgery, the risk of exposure to the ionizing radiation has increased for the orthopedic surgeons [1, 2]. Fluoroscopic procedures have been beneficial for patients undergoing orthopedic surgery because these minimally invasive procedures provide better direct visualization for soft tissue dissection, spare blood supply, and result in fewer complications than open surgeries [3, 4]. However, during the fluoroscopic procedures, the surgeons may be exposed to the primary beam and scattered radiation [2, 5]. As the use of fluoroscopy is continuously increasing in orthopedic surgery and orthopedic surgeons are often closely exposed to the radiation source during operations, they are considered a high-risk group of occupational radiation exposure [6–10].

A few epidemiologic studies have reported an increased risk of cancer from radiation exposure among orthopedic surgeons worldwide [11–13]. Diverse harmful effects, such as cancer, cataracts, chromosomal abnormalities, and other chronic diseases, have also been reported in physicians performing FGI procedures globally [14]. In addition, orthopedic surgeons have concerns about the hazards of radiation, but they have been shown to demonstrate lower rates of wearing personal protective equipment and dosimeters [15, 16]. However, previous studies on orthopedic surgeons were mainly limited by the small sample sizes and limited information on the FGI procedure-related work practices and occupational radiation exposure.

Identifying the occupational characteristics and radiation exposure could provide scientific evidence and serve as a fundamental step in developing strategies to protect against occupational radiation exposure. Therefore, this study aimed to investigate the work practices related to radiation exposure among orthopedic surgeons in South Korea.

## Methods

### Study population

We conducted a field survey using a self-administered questionnaire among orthopedic surgeons at the conference of the Korean Orthopedic Association in October 2017. The association includes all member orthopedic surgeons, and the conference is the representative meeting for orthopedic surgeons in South Korea. A total of 513 orthopedic surgeons participated in this study. Written informed consent was voluntarily obtained from all study participants prior to enrollment. The protocols of the study were reviewed and approved by the Institutional Review Board of our university (KU-IRB-17-36-A-2).

### Questionnaire and dosimetry

A detailed questionnaire was developed from a previous study for interventional medical radiation workers [17]. The questionnaire included demographics (date of birth, gender, workplace address), work history (job title, specialty, years since beginning work, total duration of work), work practices (proportion of interventional procedures performed for the recent year, working days per month, working hours per week, name of the main procedure performed, badge wearing, wearing protective equipment), and concern for developing radiation-associated diseases (5-point Likert scale). The questionnaire is provided as a supplementary material (Supplementary Table 1).

The Korea Center for Disease Control and Prevention (KCDC) has been monitoring the hospital personnel exposed to radiation since 1996; it maintains a centralized National Dosimetry Registry (NDR) and implements a lifelong follow-up management system for radiation dose using a personal thermoluminescent dosimeter (TLD) [18]. The NDR has collected dose measurements quarterly by five personnel monitoring centers designated by the KCDC for all diagnostic radiation workers. The standard protocol of wearing the NDR badge for all diagnostic radiation workers is wearing one TLD badge beneath the apron on the left side of the chest. To evaluate the individual radiation dose, the survey data were linked with the NDR up to the second quarter of 2017 with respect to the participant’s name, gender, date of birth, and workplace address. This effective dose measured in Sievert was derived from the personal dose equivalent at a depth of 10 mm (Hp [10]). The lowest detectable quarterly level of the NDR is 0.01 mSv. In cases where the dose was below the minimum detectable level, the dose was considered as half of the detectable level owing to the highly skewed distribution [19].

### Data analysis

Study participants were classified by job specialty as spine or trauma specialists (ST), other orthopedic specialists (Others), and residents, based on previous studies in which spine or trauma surgeons were reported to be exposed to radiation more than the other orthopedic surgeons [3, 6]. Descriptive statistics for the demographics and work-related characteristics are presented. The level of concern for developing radiation-associated diseases was re-categorized as low (very unlikely and unlikely), medium, and high (likely and very likely). Using the chi-square analysis, the three job specialty groups were compared according to the demographics, occupational characteristics, such as involvement with fluoroscopy, and wearing the badges and protective devices. Multivariable logistic regression analysis was used to evaluate the risk factors for the orthopedic surgeons who were not linked with the dosimetry data after adjusting for age, location of medical facility, and job specialty. Among the orthopedic physicians who were linked with the TLD data, the individual quarterly badge doses recorded during the study period were summed and divided by the number of years to obtain the annual effective doses; this was compared according to the job specialty using one-way analysis of variance (ANOVA). STATA version 14.0 (StataCorp, College Station, TX, USA) was used for statistical analysis, and *p*-values < 0.05 were considered statistically significant.

## Results

A total of 90 ST, 199 Others, and 224 residents participated in this study (Table 1). Most orthopedic surgeons were aged < 40 years and worked at general hospitals; many were young residents who were in training. The rates of high concern for occupational radiation risk were higher among the ST than the other groups. Our study participants comprised 7.1% of all the members of the Korean Orthopedic Association, but the residents in our study accounted for 23.4% of all the members of the association (Supplementary Table 2).

**Table 1:** Characteristics of the orthopedic surgeons according to the job specialty in South Korea

Characteristics	Total (n = 513) N^a^ (%)	Specialists		Residents (*n* = 224) N (%)	*p*-value^b^
		ST (*n* = 90) N (%)	Others (*n* = 199) N (%)		
Age group (year)					
< 40	403 (78.6)	55 (61.1)	125 (62.8)	223 (99.6)	< 0.001
40–49	57 (11.1)	16 (17.8)	40 (20.1)	1 (0.4)	
≥ 50	53 (10.3)	19 (21.1)	34 (17.1)	0 (0.0)	
Type of the medical facility					
General hospital	489 (95.3)	79 (87.8)	186 (93.5)	224 (100.0)	< 0.001
Small hospital	19 (3.7)	7 (7.7)	12 (6.0)	0 (0.0)	
Location of the medical facility					
Metropolitan	340 (66.3)	56 (62.2)	119 (59.8)	165 (73.7)	0.106
Province	171 (33.3)	32 (35.6)	79 (39.7)	59 (26.3)	
Level of concern for occupational radiation exposure					
Low	119 (23.2)	22 (25.3)	53 (27.9)	44 (20.7)	0.148
Medium	185 (36.1)	25 (28.7)	72 (37.9)	88 (41.3)	
High	186 (36.3)	40 (46.0)	65 (34.2)	81 (38.0)	

*ST* spine or trauma specialists; Others = other orthopedic specialists

^a^Numbers may not reflect the total owing to missing values

^b^p-value for the chi-square test or Fisher’s exact test

Approximately 40% of the orthopedic surgeons spent more than 50% of their time working with the FGI procedures when compared with their overall work (Table 2). The average work duration of performing FGI procedures was 8.0 years, and the average frequency of interventions was every 12.3 days per month. The specialists worked with fluoroscopy for longer periods than the residents; however, within the same period (i.e., workload per week or month), the residents group performed fluoroscopy procedures more frequently than the specialists. The proportion of subjects who always wore protective gears (lead aprons, thyroid shields, lead glasses, and gloves) ranged from 3 to 52%, and the residents wore the protective gears less frequently than the other specialists.

**Table 2:** Occupational characteristics of the orthopedic surgeons according to the job specialty in South Korea

Occupational characteristics	Total (n = 513) N^a^ (%)	Specialists		Residents (n = 224) N (%)	p-value^b^
		ST (n = 90) N (%)	Others (n = 199) N (%)		
**Fluoroscopy work**					
Calendar year began working with fluoroscopy					
< 1996	39 (7.6)	14 (15.6)	25 (12.6)	0 (0.0)	< 0.001
1996–2000	31 (6.0)	8 (8.9)	22 (11.1)	1 (0.4)	
2001–2005	33 (6.4)	10 (11.1)	23 (11.6)	0 (0.0)	
2006–2010	115 (22.4)	31 (34.4)	73 (36.7)	11 (4.9)	
2011–2015	247 (48.1)	24 (26.7)	53 (26.6)	170 (76.2)	
≥ 2016	47 (9.2)	3 (3.3)	3 (1.5)	41 (18.4)	
Years working with fluoroscopy					
< 5	193 (37.6)	16 (17.8)	9 (4.5)	168 (75.0)	< 0.001
5–9	197 (38.4)	38 (42.2)	103 (52.0)	56 (25.0)	
≥ 10	122 (23.8)	36 (40.0)	86 (43.4)	0 (0.0)	
Proportion time spent working with fluoroscopy					
100%	5 (1.0)	2 (2.2)	1 (0.5)	2 (0.9)	< 0.001
75–99%	49 (9.6)	10 (11.1)	11 (5.6)	28 (12.7)	
50–74%	152 (29.6)	28 (31.1)	44 (22.2)	80 (36.4)	
25–49%	161 (31.4)	26 (28.9)	60 (30.3)	75 (34.1)	
< 25%	141 (27.5)	24 (26.7)	82 (41.4)	35 (15.9)	
Working days per month with fluoroscopy					
< 10	163 (31.8)	26 (29.2)	102 (52.3)	35 (15.7)	< 0.001
10–15	212 (41.3)	48 (53.9)	78 (40.0)	86 (38.6)	
≥ 16	132 (25.7)	15 (16.9)	15 (7.7)	102 (45.7)	
Number of fluoroscopic procedures per week					
< 5	152 (29.6)	19 (21.3)	83 (42.1)	50 (22.5)	< 0.001
5–10	221 (43.1)	49 (55.1)	84 (42.6)	88 (39.6)	
≥ 11	135 (26.3)	21 (23.6)	30 (15.2)	84 (37.8)	
Working hours per week with fluoroscopy					
< 6	185 (36.1)	37 (41.6)	98 (50.0)	50 (22.5)	< 0.001
6–12	155 (30.2)	27 (30.3)	60 (30.6)	68 (30.6)	
≥ 13	167 (32.6)	25 (28.1)	38 (19.4)	104 (46.8)	
**Badge wearing**					
Provided with the badges regularly					
No	360 (70.2)	58 (64.4)	139 (70.9)	163 (72.8)	0.340
Yes	150 (29.2)	32 (35.6)	57 (29.1)	61 (27.2)	
Proportion of personnel wearing the badges					
100%	20 (13.3)	7 (21.9)	8 (14.0)	5 (8.2)	0.323
75–99%	21 (14.0)	4 (12.5)	11 (19.3)	6 (9.8)	
25–74%	44 (29.3)	9 (28.1)	18 (31.6)	17 (27.9)	
1–24%	36 (24.0)	8 (25.0)	12 (21.1)	16 (26.2)	
0%	29 (19.3)	4 (12.5)	8 (14.0)	17 (27.9)	
**Personal protective equipment use**					
Proportion of personnel wearing the lead aprons					
100%	269 (52.4)	55 (61.1)	106 (53.3)	108 (48.2)	0.414
75–99%	147 (28.7)	19 (21.1)	53 (26.6)	75 (33.5)	
25–74%	59 (11.5)	9 (10.0)	22 (11.1)	28 (12.5)	
1–24%	20 (3.9)	4 (4.4)	10 (5.0)	6 (2.7)	
0%	18 (3.5)	3 (3.3)	8 (4.0)	7 (3.1)	
Proportion of personnel wearing the thyroid shields					
100%	150 (29.2)	39 (43.3)	62 (31.3)	49 (21.9)	< 0.001
75–99%	107 (20.9)	13 (14.4)	40 (20.2)	54 (24.1)	
25–74%	82 (16.0)	9 (10.0)	22 (11.1)	51 (22.8)	
1–24%	51 (9.9)	9 (10.0)	17 (8.6)	25 (11.2)	
0%	122 (23.8)	20 (22.2)	57 (28.8)	45 (20.1)	
Proportion of personnel wearing the lead glasses					
100%	18 (3.5)	9 (10.1)	7 (3.6)	2 (0.9)	< 0.001
75–99%	10 (1.9)	3 (3.4)	5 (2.6)	2 (0.9)	
25–74%	18 (3.5)	6 (6.7)	6 (3.1)	6 (2.7)	
1–24%	29 (5.7)	6 (6.7)	10 (5.1)	13 (5.8)	
0%	434 (84.6)	65 (73.0)	168 (85.7)	201 (89.7)	
Proportion of personnel wearing the lead gloves					
100%	13 (2.5)	9 (10.0)	2 (1.0)	2 (0.9)	< 0.001
75–99%	8 (1.6)	3 (3.3)	3 (1.5)	2 (0.9)	
25–74%	16 (3.1)	4 (4.4)	6 (3.0)	6 (2.7)	
1–24%	24 (4.7)	7 (7.8)	8 (4.1)	9 (4.0)	
0%	450 (87.7)	67 (74.4)	178 (90.4)	205 (91.5)	

*ST* spine or trauma specialists; Others = other orthopedic specialists

^a^Numbers may not reflect the total owing to missing values

^b^p-value for the chi-square test or Fisher’s exact test

Among the 513 orthopedic surgeons who responded to the survey, only 121 (23.6%) were linked with the TLD data (Table 3). The odds ratios (OR) of not being linked with the dosimetry data was significantly increased among the surgeons working at small hospitals (OR 4.76, 95% confidence interval [CI] 1.05–21.51) and residents group (OR 2.10, 95% CI 1.11–3.95) after adjusting for potential confounding factors. Among the orthopedic doctors who were linked with the dosimetry data, the annual effective dose was higher in the ST (0.20 mSv) than in Others (0.11 mSv) or residents (0.09 mSv) (Supplementary Table 3).

**Table 3:** Odds ratios of not being linked with dosimetry data among the orthopedic surgeons in South Korea

Characteristics	Not linked with the dosimetry (*n* = 392) N^a^ (%)	Linked with the dosimetry (*n* = 121) N (%)	OR	95% CI		OR^b^	95% CI	
Age group (year)								
< 40	320 (81.6)	83 (68.6)	2.34	1.28	4.28	1.72	0.89	3.33
40–49	39 (9.9)	18 (14.9)	1.31	0.60	2.89	1.41	0.63	3.16
≥ 50	33 (8.4)	20 (16.5)	1.00	reference		1.00	reference	
Type of medical facility								
General hospital	371 (94.6)	119 (98.3)	1.00	reference		1.00	reference	
Small hospital	17 (4.3)	2 (1.6)	2.72	0.62	11.97	4.76	1.05	21.51
Location of medical facility								
Metropolitan	265 (67.9)	75 (62.0)	1.00	reference		1.00	reference	
Province	125 (32.1)	46 (38.0)	0.77	0.50	1.18	0.89	0.57	1.38
Level of concern								
Low	88 (23.6)	31 (26.5)	1.00	reference		1.00	reference	
Medium	143 (38.3)	42 (35.9)	1.20	0.70	2.05	1.09	0.62	1.93
High	142 (38.1)	44 (37.6)	1.14	0.67	1.93	0.95	0.53	1.67
Job specialty								
ST	63 (16.1)	27 (22.3)	1.00	reference		1.00	reference	
Others	141 (36.0)	58 (47.9)	1.04	0.60	1.80	1.11	0.63	1.95
Residents	188 (48.0)	36 (29.8)	2.24	1.26	3.98	2.10	1.11	3.95
Calendar year began working with fluoroscopy								
< 1996	23 (5.9)	16 (13.2)	1.00	reference		1.00	reference	
1996–2000	19 (4.8)	12 (9.9)	1.10	0.42	2.89	1.10	0.31	3.88
2001–2005	25 (6.4)	8 (6.6)	2.17	0.78	6.03	2.82	0.69	11.55
2006–2010	82 (20.9)	33 (27.3)	1.73	0.81	3.68	2.08	0.50	8.61
2011–2015	206 (52.6)	41 (33.9)	3.50	1.70	7.19	3.13	0.72	13.60
≥ 2016	37 (9.4)	11 (9.1)	2.34	0.93	5.92	2.20	0.44	11.03
Years working with fluoroscopy								
< 5	160 (40.8)	33 (27.3)	2.70	1.60	4.57	1.85	0.74	4.64
5–9	153 (39.0)	44 (36.4)	1.94	1.18	3.19	1.59	0.75	3.37
≥ 10	79 (20.2)	44 (36.4)	1.00	reference		1.00	reference	
Proportion time spent working with fluoroscopy								
< 25%	105 (26.8)	36 (29.8)	1.00	reference		1.00	reference	
25–49%	117 (29.8)	44 (36.4)	0.95	0.57	1.59	0.76	0.44	1.31
50–74%	120 (30.6)	32 (26.4)	1.29	0.75	2.21	1.03	0.58	1.85
75–99%	40 (10.2)	9 (7.4)	1.52	0.67	3.45	1.17	0.49	2.78
100%	5 (1.3)	0 (0.0)	–			–		
Proportion of personnel wearing the badges								
0%	291 (74.2)	48 (39.7)	2.60	0.95	7.09	2.08	0.74	5.88
1–24%	50 (12.8)	29 (24.0)	0.74	0.26	2.13	0.61	0.20	1.81
25–74%	24 (6.1)	30 (24.8)	0.34	0.11	1.03	0.27	0.09	0.85
75–99%	13 (3.3)	8 (6.6)	0.70	0.19	2.56	0.57	0.15	2.17
100%	14 (3.6)	6 (5.0)	1.00	reference		1.00	reference	

*OR* odds ratio, *AOR* adjusted odds ratio, *ST* spine or trauma specialists, *Others* other orthopedic specialists, *CI* confidence intervals

^a^Numbers may not reflect the total owing to missing values

^b^Adjusted for age, location of medical facility, and job specialty

## Discussion

The orthopedic surgeons in this study often performed fluoroscopy during the course of their work; however, the rate of wearing the dosimetry badges and protective devices has been shown to be low. In addition, most participants in this study were not linked to the national dosimetry data. The residents group experienced more unfavorable working conditions in terms of radiation exposure than the other specialists. Among the orthopedic surgeons who were linked with the dosimetry data, the ST had higher radiation doses than the other orthopedic surgeons. To the best of our knowledge, this study is the first attempt to investigate the status of occupational radiation exposure among the orthopedic surgeons in South Korea. Our findings may contribute to the increasing awareness of the radiation protection and its potential risks among hospital workers.

The rates of use of fluoroscopy and protective devices in our study were comparable with those in a worldwide study on orthopedic surgeons that reported that more than half of the procedures performed by 61.5% of the surgeons involved radiation exposure, whereas the rates of using lead aprons, thyroid shields, and lead glasses were 65, 30.8, and 2.5%, respectively [16]. According to a US survey, 50% of the subjects reported that lead aprons were not available and the remaining half reported that they were not appropriately sized [20]. The other study reported that one out of three orthopedic surgery residents were not provided protective gowns in the U.S. [21]. These rates of personal protective equipment use were lower among the orthopedic surgeons than among the interventional cardiologists; particularly, the rates of wearing the lead aprons, thyroid protectors, and lead glasses were 100, 93, and 18%, respectively [22]. The possible reasons for low rates of apron use was not being properly provided with aprons and inconvenience while wearing aprons among the Korean Intern and Residents Association [23].

The rate of always wearing a badge was generally comparable with those in a worldwide survey that reported that about one-fifth of the orthopedic surgeons wear a dosimeter [16]. The Irish orthopedic surgeons also reported that the regular use of dosimeters among the orthopedic trainees was 15% [24], and only 5% of the orthopedic surgeons were reported to wear the TLD during surgery in Turkey [10]. A possible reason for the orthopedic surgeons not preferring to wear the dosimeters is that they may believe that it will affect their performance and make them uncomfortable [20, 25]. Another reason may be that if their radiation exposure was greater than the specified limit, they would be prohibited from operating with fluoroscopy for a specified period of time; therefore, they do not routinely wear the personal dosimetry badges [26].

Approximately three-fourth of our participants were not linked with the national dosimetry data, and the risk of not being linked was increased among the residents group and those who worked at smaller medical facilities. A possible reason may be owing to the incompleteness by which the radiation safety managers at each medical facility select the radiation exposed orthopedic surgeons. In 2018, 730 members of the Korean Intern and Resident Association responded that 69.3% of them had been exposed to fluoroscopy; however, only 8.8% of them wore the TLD when exposed to radiation during fluoroscopy [23]. These results suggest that the monitoring system for the orthopedic surgeons with radiation exposure appears to be unsuccessful—in particular for residents and for small hospital workers—and additional efforts to improve dosimetry monitoring system are required in South Korea.

The doses monitored in the dosimetry data of the orthopedic surgeons in this study were lower than those reported in Italy [13], The Philippines [27], India [28], and South Korea [29, 30]. This finding supports the notion that the radiation exposure among the orthopedic surgeons may be widely ranged, depending on the work procedures and experience [3]. The higher radiation dose among ST in this study may be attributed to longer exposure time and more fluoroscopic shots in ST [7–10] than those of other surgeons. However, the actual exposure might be underestimated because of the orthopedic surgeons’ low rate of wearing the badges and protective devices. Owing to the irregular and inconsistent use of dosimeters, estimating the radiation dose with personal dosimeters needs to be improved among orthopedic surgeons.

More than one-third of the orthopedic surgeons were greatly concerned about health problems caused by occupational radiation exposure, which is similar to that reported in a previous worldwide study on orthopedic surgeons [16]. Among all the participants, the ST surgeons showed a higher rate of concern than the other groups, and this may be related to them performing a higher proportion of the FGI procedures than other doctors. Previously, a lack of radiation knowledge and awareness about fluoroscopy were also associated with a high level of concern and low rate of wearing the protective devices [15, 16]; therefore, education about radiation exposure is warranted for orthopedic surgeons.

This study is the first attempt to investigate the status of occupational radiation exposure in a relatively large number of orthopedic surgeons in South Korea. However, the study did not represent all members of the Korean Orthopedic Association, although the residents group may represent the total population of the orthopedic residents. The high proportion of residents in this survey may be owing to their high participation in the conference and having greater interests in radiation exposure because of their harsh working conditions. Our findings represent mainly male orthopedic surgeons owing to a very small number of female participants. In addition, this study recruited few orthopedic surgeons who worked at clinics, which limited our findings mainly to orthopedic surgeons at large hosptials. Recall bias may be present because of using a self-administered questionnaire. However, the participants were a relatively young and highly educated group and the questionnaire items were related to their daily work; therefore, the bias should be minimal. The information on self-reported working practices regarding radiation exposure has been reported as reliable among South Korean radiologic technologists [31].

## Conclusions

We reported the occupational characteristics and radiation exposure among orthopedic surgeons in South Korea. Although many orthopedic surgeons perform interventional FGI procedures, unfavorable work characteristics—such as the low rate of wearing protective devices and dosimeters—may increase the radiation risk. Additionally, badge monitoring was not noted for the relatively large proportion of the orthopedic surgeons who performed FGI procedures. The orthopedic surgeons are at a risk of occupational radiation exposure, and more intensive approaches are needed to reduce radiation exposure and protect possible work-related health effects.

## Supplementary information

**Additional file 1: Supplementary Table 1.** Questionnaire used for orthopedic surgeons at the conference of the Korean Orthopedic Association in 2017.

**Additional file 2: Supplementary Table 2.** Comparison of selected characteristics between the target population and the study participants.

**Additional file 3: Supplementary Table 3.** Annual effective doses by occupational characteristics and specialties among orthopedic surgeons in South Korea.

## Data Availability

The datasets generated during the current study are not publicly available because the detailed individual data is restricted for both legal and ethical concerns but are available from the corresponding author on reasonable request.

## Abbreviations

NDR: National Dosimetry Registry
TLD: Thermoluminescent dosimeter
KCDC: Korea Centers for Disease Control and Prevention
ST: Spine or trauma specialists
Others: Other orthopedic specialists
OR: Odds ratio
CI: Confidence intervals
