# Supplementary material for: Radiation exposure and fluoroscopically-guided interventional procedures among orthopedic surgeons in South Korea
Source: J Occup Med Toxicol. 2020 Aug 11;15:24. doi: 10.1186/s12995-020-00276-x (PMC7418415; doi:10.1186/s12995-020-00276-x)
Supplement: Supplementary file 1 — Additional file 1: Supplementary Table 1. Questionnaire used for orthopedic surgeons at the conference of the Korean Orthopedic Association in 2017. [file 12995_2020_276_MOESM1_ESM.docx]

Supplementary Table 1. Questionnaire used for orthopedic surgeons at the conference of the Korean Orthopedic Association in 2017

| **1. What is your occupation?** | | | | | |
| --- | --- | --- | --- | --- | --- |
| ① Specialist  ② General practitioner  ③ Radiologic technologist  ④ Nurse  ⑤ Medical laboratory technologist  ⑥ Other (Occupation: ) | | | | | |
|  | | | | | |
| **2. What is your sub-specialty? (multiple selection possible)** | | | | | |
| ① Spine  ② Joint  ③ Trauma  ④ Hand and Upper extremity  ⑤ Foot and ankle surgery  ⑥ Orthopedic oncologist  ⑦ Pediatric orthopedic  ⑧ Other (subspecialty: )  **3. What year did you start fluoroscopically-guided procedures including C-arm fluoroscopic X-ray?**   \| **Y** \| **Y** \| **Y** \| **Y** \|  \| \| --- \| --- \| --- \| --- \| --- \|   **4. How many YEARS did you perform with fluoroscopically-guided procedures?**   \| **Y** \| **Y** \|  \| \| --- \| --- \| --- \|   **5. What is the proportion of fluoroscopically-guided procedures in your entire work in the past year?**  ① 100% ② 75-99% ③ 50-74% ④ 25-49% ⑤ less than 25%  **6. During the past year, how many days per month did you perform fluoroscopically-guided procedures?**   \|  \| **D** \| **D** \|  \| \| --- \| --- \| --- \| --- \|   **7. During the past year, how many times and hours per week did you usually perform with**  **fluoroscopically-guided procedures?**   \|  \|  \|  \| **Times/Week** \| \| --- \| --- \| --- \| --- \|  \|  \|  \|  \| **Hours/Week** \| \| --- \| --- \| --- \| --- \|     **8. What kind of fluoroscopically-guided procedures have you participated in the most in the last year?**   \| **Procedure ( )** \| \| --- \|   **9. Do you regularly receive a dosimeter?**  ① No ② Yes  **10. When performing fluoroscopy procedures, what percentage of the time did you wear a dosimeter?**  ① 100% ② 75-99% ③ 25-74% ④ 1-24% ⑤ 0%  **11. When performing fluoroscopic procedures, where do you usually wear your dosimetry badge?**  ① Under the apron ② Outside the apron ③ Other ( ) ④ Do not usually wear a badge | | | | | |
|  | | | | | |
| **12. When performing fluoroscopy procedures, what percentage of the time did you wear protective measures?** | **100%** | **75-99%** | **25-74%** | **1-24%** | **0%** |
| **Lead apron** | ① | ② | ③ | ④ | ⑤ |
| **Thyroid shield** | ① | ② | ③ | ④ | ⑤ |
| **Lead glasses** | ① | ② | ③ | ④ | ⑤ |
| **Leaded gloves** | ① | ② | ③ | ④ | ⑤ |
| **Mobile floor shield** | ① | ② | ③ | ④ | ⑤ |
| **Ceiling suspended shield** | ① | ② | ③ | ④ | ⑤ |
|  | | | | | |

| **13. While working in the field of fluoroscopy, were you ever asked to discontinue working as an orthopedic surgeon for a while because your radiation exposure exceeded the allowable limit?** |
| --- |
| ① No ② Yes ③ Do not know |
|  |
| **14. Has your white blood cell count been depressed below normal as a result of working in the field of fluoroscopy?** |
| ① No ② Yes ③ Do not know ④ Never had a medical check up |
| **15. What is your current status in relation to smoking cigarettes?** |
| ① Never a smoker ② quit smoking ③ currently a smoker |
| **16.** **How many alcoholic beverages (beer, wine, or liquor) have you usually had in the last year?** |
| ① Never drink ② Less than once a month ③ 2-4 drinks a month  ④ 2-3 drinks a week ⑤ More than 4 drinks a week |
|  |
| **17. Have you ever had the following personal diagnostic radiation exams or personal therapeutic radiation procedures?** |

| **Exam/Procedure** | **Yes** | **No** | **Exam/Procedure** | **Yes** | **No** | **Exam/Procedure** | **Yes** | **No** |
| --- | --- | --- | --- | --- | --- | --- | --- | --- |
| **CT scan** | ① | ② | **Routine fluoroscopy**  **(upper GI series, Urethrogram, etc.)** | ① | ② | **Nuclear medicine exams or therapy**  **(Thyroid scan, SPECT, etc.)** | ① | ② |
| **PET scan** | ① | ② | **Fluoroscopy** | ① | ② | **Radiotherapy** | ① | ② |

| **18. Have you ever had any of the following medical conditions after performing fluoroscopic procedures?** |
| --- |

| **Disease/Symptom** | **Yes** | **No** | **Disease/Symptom** | **Yes** | **No** | **Disease/Symptom** | **Yes** | **No** | |
| --- | --- | --- | --- | --- | --- | --- | --- | --- | --- |
| **Cataracts** | ① | ② | **Ocular inflammation** | ① | ② | **Anemia** | ① | | ② |
| **Hypertension** | ① | ② | **Hypercholesterolemia** | ① | ② | **Malignant neoplasms** | ① | | ② |
| **Benign thyroid tumor** | ① | ② | **Hyperthyroidism** | ① | ② | **Hypothyroidism** | ① | | ② |
| **Neck/back pain** | ① | ② | **Skin damage (inflammation)** | ① | ② | **Radiation-induced alopecia** | 1. ①① | | ②② |

| \| **19. How likely are you to develop a**  **Very unlikely ← ( ① - ② - ③ - ④ - ⑤ ) → Very likely**  **disease from your occupational radiation**  **exposure?** \| \| --- \| |
| --- | --- |
| \| **Sex** \| ① Male ② Female \| **Date of birth** \| \| \| M \| M \|  \| D \| D \|  \| Y \| Y \|  \| \| --- \| --- \| --- \| --- \| --- \| --- \| --- \| --- \| --- \| \| \| \| --- \| --- \| --- \| --- \| --- \| --- \| --- \| --- \| --- \| --- \| --- \| --- \| --- \| --- \| --- \| \| **Working**  **area** \| **City/County/District** \| **Name of medical institution** \|  \| **Phone number** \|  \| |
|  |
