# Supplementary material for: Radiation exposure and fluoroscopically-guided interventional procedures among orthopedic surgeons in South Korea
Source: J Occup Med Toxicol. 2020 Aug 11;15:24. doi: 10.1186/s12995-020-00276-x (PMC7418415; doi:10.1186/s12995-020-00276-x)
Supplement: Supplementary file 2 — Additional file 2: Supplementary Table 2. Comparison of selected characteristics between the target population and the study participants. [file 12995_2020_276_MOESM2_ESM.docx]

| Supplementary Table 2. Comparison of selected characteristics between the target population and the study participants | | | | | | | | |
| --- | --- | --- | --- | --- | --- | --- | --- | --- |
| Characteristics | Target population^a^ | | |  | Study participants | | | |
|  | Total (n=7249)  N^b^ (%) | Male (n=7195)  N (%) | Female (n=54)  N (%) | | Total (n=513) N (%) | | Male (n=509)  N (%) | Female (n=4)  N (%) |
| Job specialty |  |  |  |  |  |  | |  |
| Specialists | 6275 (86.6) | 6275 (87.2) | 40 (74.1) |  | 288 (56.1) | 286 (56.2) | | 2 (50.0) |
| Residents | 960  (13.2) | 920  (12.8) | 14 (25.9) |  | 225 (43.9) | 223 (43.8) | | 2 (50.0) |
| Age group (year) |  |  |  |  |  |  | |  |
| <30 | 391  (5.4) | 386  (5.4) | 5  (9.3) |  | 41  (8.0) | 41  (8.1) | | 0  (0.0) |
| 30–39 | 2246 (31.0) | 2214 (30.8) | 32 (59.3) |  | 361 (70.5) | 358 (70.5) | | 3 (75.0) |
| 40–49 | 1950 (26.9) | 1938 (26.9) | 12 (22.2) |  | 57  (11.1) | 56  (11.0) | | 1 (25.0) |
| ≥50 | 2662 (36.7) | 2657 (36.9) | 5  (9.3) |  | 53  (10.4) | 53  (10.4) | | 0  (0.0) |
| Type of medical facility | |  |  |  |  |  | |  |
| General hospital | 4203 (58.0) | 4158 (57.8) | 45 (83.3) |  | 484 (95.5) | 480 (95.4) | | 4 (100.0) |
| Hospital | 747  (10.3) | 743  (10.3) | 4  (7.4) |  | 9  (1.8) | 9  (1.8) | | 0  (0.0) |
| Clinic | 2177 (30.0) | 2172 (30.2) | 5  (9.3) |  | 4  (0.8) | 4  (0.8) | | 0  (0.0) |
| Public health center | 57  (0.8) | 57  (0.8) | 0  (0.0) |  | 10  (2.0) | 10  (2.0) | | 0  (0.0) |
| Long-term care hospital | 65  (0.9) | 65  (0.9) | 0  (0.0) |  | 0  (0.0) | 0  (0.0) | | 0  (0.0) |
| Location of the medical facility | | |  |  |  |  | |  |
| Metropolitan | 4125 (56.9) | 4089 (56.8) | 36 (66.7) |  | 340 (66.7) | 338 (66.8) | | 2 (50.0) |
| Province | 3124 (43.1) | 3106 (43.2) | 18 (33.3) |  | 170 (33.3) | 168 (33.2) | | 2 (50.0) |
| ^a^Participants registered with the Korean Orthopedic Association as of 2018. | | | | | | | | |
| ^b^Numbers may not reflect the total owing to missing values. | | | | | |  | |  |
