# Supplementary material for: Radiation exposure and fluoroscopically-guided interventional procedures among orthopedic surgeons in South Korea
Source: J Occup Med Toxicol. 2020 Aug 11;15:24. doi: 10.1186/s12995-020-00276-x (PMC7418415; doi:10.1186/s12995-020-00276-x)
Supplement: Supplementary file 3 — Additional file 3: Supplementary Table 3. Annual effective doses by occupational characteristics and specialties among orthopedic surgeons in South Korea. [file 12995_2020_276_MOESM3_ESM.docx]

| Supplementary Table 3. Annual effective doses by occupational characteristics and specialties among the orthopedic surgeons in South Korea^*^ | | | | | | | | | |
| --- | --- | --- | --- | --- | --- | --- | --- | --- | --- |
| Occupational characteristics | Annual effective doses (mSv) | | | | | | | | |
|  | Total | | ST | | Others | | Residents | | p-value^a^ |
| Total | 121 | 0.12 ± 0.24 | 27 | 0.20 ± 0.43 | 58 | 0.11 ± 0.16 | 36 | 0.09 ± 0.10 | 0.618 |
| Age group (year) | | |  |  |  |  |  |  |  |
| <40 | 83 | 0.09 ± 0.12 | 13 | 0.11 ± 0.16 | 34 | 0.08 ± 0.12 | 36 | 0.09 ± 0.10 | 0.758 |
| 40–49 | 18 | 0.22 ± 0.50 | 7 | 0.41 ± 0.79 | 11 | 0.11 ± 0.14 | - | - | 0.329 |
| ≥50 | 20 | 0.19 ± 0.22 | 7 | 0.17 ± 0.21 | 13 | 0.20 ± 0.23 | - | - | 0.838 |
| p-value^b^ | | 0.016 |  | 0.144 |  | 0.071 |  | - |  |
| Type of medical facility^c^ | | | |  |  |  |  |  |  |
| General hospital | 119 | 0.12 ± 0.24 | 25 | 0.21 ± 0.45 | 58 | 0.11 ± 0.16 | 36 | 0.09 ± 0.10 | 0.742 |
| Location of medical facility | | | | |  |  |  |  |  |
| Metropolitan | 75 | 0.10 ± 0.12 | 16 | 0.14 ± 0.17 | 32 | 0.07 ± 0.08 | 27 | 0.10 ± 0.11 | 0.08 |
| Province | 46 | 0.17 ± 0.35 | 11 | 0.32 ± 0.67 | 26 | 0.17 ± 0.21 | 9 | 0.04 ± 0.05 | 0.132 |
| p-value^a^ | | 0.981 |  | 0.603 |  | 0.073 |  | 0.011 |  |
| Calendar year began working with fluoroscopy | | | | | | |  |  |  |
| <1996 | 16 | 0.12 ± 0.16 | 5 | 0.18 ± 0.24 | 11 | 0.10 ± 0.10 | - | - | 0.338 |
| 1996-2010 | 53 | 0.17 ± 0.33 | 18 | 0.25 ± 0.50 | 32 | 0.13 ± 0.19 | 3 | 0.13 ± 0.12 | 0.611 |
| ≥2011 | 52 | 0.08 ± 0.11 | 4 | 0.01 ± 0.01 | 15 | 0.09 ± 0.14 | 33 | 0.08 ± 0.10 | 0.179 |
| p-value^b^ | | 0.131 |  | 0.054 |  | 0.622 |  | - |  |
| Years working with fluoroscopy | | | | |  |  |  |  |  |
| <5 | 33 | 0.10 ± 0.12 | 4 | 0.14 ± 0.19 | 1 | 0.02 | 28 | 0.09±0.11 | 0.840 |
| 5–9 | 44 | 0.09 ± 0.11 | 10 | 0.12 ± 0.18 | 26 | 0.08 ± 0.09 | 8 | 0.07 ± 0.07 | 0.969 |
| ≥10 | 44 | 0.18 ± 0.36 | 13 | 0.29 ± 0.59 | 31 | 0.13 ± 0.19 | - | - | 0.234 |
| p-value^b^ | | 0.405 |  | 0.359 |  | 0.555 |  | - |  |
| Proportion working with fluoroscopy | | | | | |  |  |  |  |
| 50–100% | 41 | 0.20 ± 0.36 | 12 | 0.37 ± 0.61 | 11 | 0.19 ± 0.19 | 18 | 0.10 ± 0.10 | 0.352 |
| 25–49% | 44 | 0.06 ± 0.07 | 7 | 0.06 ± 0.08 | 24 | 0.06 ± 0.08 | 13 | 0.04 ± 0.04 | 0.981 |
| <25% | 36 | 0.12 ± 0.17 | 8 | 0.07 ± 0.06 | 23 | 0.163 ± 0.20 | 5 | 0.15 ± 0.19 | 0.921 |
| p-value^b^ | | 0.075 |  | 0.219 |  | 0.175 |  | 0.762 |  |
| Working days per month with fluoroscopy | | | | | | |  |  |  |
| <10 | 37 | 0.11 ± 0.15 | 7 | 0.07 ± 0.07 | 26 | 0.12 ± 0.17 | 4 | 0.08 ± 0.12 | 0.787 |
| 10–15 | 57 | 0.13 ± 0.31 | 16 | 0.25 ± 0.54 | 27 | 0.09 ± 0.15 | 14 | 0.08 ± 0.08 | 0.513 |
| ≥16 | 27 | 0.13 ± 0.16 | 4 | 0.25 ± 0.22 | 5 | 0.17 ± 0.21 | 18 | 0.10 ± 0.12 | 0.405 |
| p-value^b^ | | 0.723 |  | 0.202 |  | 0.504 |  | 0.859 |  |
| Number of fluoroscopic procedures per week | | | | | | |  |  |  |
| <5 | 32 | 0.10 ± 0.10 | 5 | 0.12 ± 0.17 | 17 | 0.09 ± 0.08 | 10 | 0.11 ± 0.10 | 0.910 |
| 5–10 | 56 | 0.16 ± 0.33 | 12 | 0.29 ± 0.62 | 32 | 0.14 ± 0.20 | 12 | 0.09 ± 0.13 | 0.707 |
| ≥11 | 33 | 0.08 ± 0.12 | 10 | 0.13 ± 0.18 | 9 | 0.04 ± 0.06 | 14 | 0.08 ± 0.08 | 0.424 |
| p-value^b^ | | 0.467 |  | 0.878 |  | 0.447 |  | 0.788 |  |
| Working hours per week with fluoroscopy | | | | | | |  |  |  |
| <6 | 43 | 0.11 ± 0.15 | 11 | 0.11 ± 0.18 | 24 | 0.12 ± 0.16 | 8 | 0.08 ± 0.09 | 0.805 |
| 6–12 | 37 | 0.18 ± 0.36 | 7 | 0.53 ± 0.75 | 22 | 0.09 ± 0.10 | 8 | 0.12 ± 0.15 | 0.116 |
| ≥13 | 41 | 0.09 ± 0.15 | 9 | 0.06 ± 0.07 | 12 | 0.13 ± 0.26 | 20 | 0.08 ± 0.09 | 0.844 |
| p-value^b^ | | 0.469 | 27 | 0.883 | 58 | 0.456 | 36 | 0.955 |  |
| AED=annual effective dose; ST=spine or trauma specialists; Others=other specialists | | | | | | | | | |
| ^*^Only participants who had dosimetry data are included.  ^a^p-value for ANOVA or the t-test | | | | | | | | | |
| ^b^p-value for the trend test | | | | | | | | | |
| ^c^Surgeons working at small hospitals were not shown due to small number. | | | | | | | | | |
|  | | | | | | | | | |
